# Reflecting the real value of health care resources in modelling and cost-effectiveness studies—The example of viral load informed differentiated care

**DOI:** 10.1371/journal.pone.0190283

**Published:** 2018-01-02

**Authors:** Paul Revill, Simon Walker, Valentina Cambiano, Andrew Phillips, Mark J. Sculpher

**Affiliations:** 1 Centre for Health Economics, University of York, York, United Kingdom; 2 Research Department of Infection & Population Health, UCL, London, United Kingdom; University of New South Wales, AUSTRALIA

## Abstract

**Background:**

The WHO HIV Treatment Guidelines suggest routine viral-load monitoring can be used to differentiate antiretroviral therapy (ART) delivery and reduce the frequency of clinic visits for patients stable on ART. This recommendation was informed by economic analysis that showed the approach is very likely to be cost-effective, even in the most resource constrained of settings. The health benefits were shown to be modest but the costs of introducing and scaling up viral load monitoring can be offset by anticipated reductions in the costs of clinic visits, due to these being less frequent for many patients.

**Key issues for economic evaluation:**

The cost-effectiveness of introducing viral-load informed differentiated care depends upon whether cost reductions are possible if the number of clinic visits is reduced and/or how freed clinic capacity is used for alternative priorities. Where freed resources, either physical or financial, generate large health gains (e.g. if committed to patients failing ART or to other high value health care interventions), the benefits of differentiated care are expected to be high; if however these freed physical resources are already under-utilized or financial resources are used less efficiently and would not be put to as beneficial an alternative use, the policy may not be cost-effective. The implication is that the use of conventional unit costs to value resources may not well reflect the latter’s value in contributing to health improvement. Analyses intended to inform resource allocated decisions in a number of settings may therefore have to be interpreted with due consideration to local context. In this paper we present methods of how economic analyses can reflect the real value of health care resources rather than simply applying their unit costs. The analyses informing the WHO Guidelines are re-estimated by implementing scenarios using this framework, informing how differentiated care can be prioritized to generate greatest gains in population health.

**Implications:**

The findings have important implications for how economic analyses should be undertaken and reported in HIV and other disease areas. Results provide guidance on conditions under which viral load informed differentiated care will more likely prove to be cost effective when implemented.

## Introduction

Economic evaluation studies (such as cost-effectiveness analyses) are now widely used to inform HIV policy decisions concerning the allocation of limited resources. Their findings are employed at various levels of governance—sometimes at the programme or country-level; at others internationally, as part of decisions affecting multiple jurisdictions. Examples of the former include the setting of national HIV investment plans,[1,2] including to support country applications to access the Global Fund for AIDS, TB and Malaria; and examples of the latter include the development of World Health Organization (WHO) HIV Treatment Guidelines[3–5] and priority setting within the US President’s Emergency Fund for AIDS Relief (PEPFAR). The decisions made affect who benefits from HIV care, the types of interventions they receive, the magnitude of health improvement; and also, as a consequence of resource constraints, who goes without care that could offer health improvement, both in HIV and in other disease areas.

Whatever the types of decisions or the administrative level to which economic analyses are aimed, it is important to recognize that to be of value they should ultimately affect positive changes at the local level—leading to benefits for real people, in real places. To be reliably informative, economic evaluation analyses should exhibit at least 2 key features. (1) They should present the anticipated health benefits, using a generic measure suitable to a wide range of competing claims upon resources (e.g. quality-adjusted or disability-adjusted life years; QALYs, DALYs), as well as their costs. (2) They should indicate whether the health benefits from any policy decision are likely to exceed the forgone benefits as a result of committed resources consequentially being unavailable for other activities (i.e. whether benefits exceed opportunity costs). In practice, this usually involves estimating, compared to alternative approaches to delivering care to a given patient group, the benefits of an intervention and the additional costs imposed. The cost per unit of benefit (e.g. QALY; DALY-averted) is then compared to a benchmark of value (e.g. a cost-effectiveness threshold) which represents the cost per unit of benefit of interventions forgone (i.e. what the resources could alternatively be used for)–if it is lower, the intervention is cost-effective; if higher, it is not.

In most cases, the type of policy problems to which economic evaluations have been applied have been conceived as being the flexible allocation of a budget—sometimes for one year, often available for use over many years—across competing priorities. However, in most countries health care is primarily a planned sector which is slow to adjust to changing circumstances and for which investments in the core inputs of delivery (e.g. in human resources and capital infrastructures) have long-term implications that are to a degree fixed and often cannot easily be changed in future.[6] In such situations, it is not evident that the unit costs used in economic evaluation studies, which typically are the long-run average costs or prices at which resources are purchased, suitably reflect the true value of resources in terms of their contributions to improving health. This is especially the case where resources are not traded on open, competitive markets that can respond effectively to price signals (such as with the employment of skilled health care workers, such as nurses) or where they are fixed assets, the prices of which are determined long in advance and where subsequent realisation of uncertainties changes their values.

It is therefore useful to consider whether economic evaluation studies, conceived around the allocation of a financial budget and using long-run average costs/prices, are always likely to recommend the allocation of resources that is likely to lead to maximum population health benefits when those resources also include real ‘non-financial’ health care inputs. If not, this is important for 2 reasons: (1) it means health systems are likely to generate less health than they are otherwise able, by following the recommendations of studies which fail to account for these non-financial resource costs—and individuals in the population will unnecessarily incur premature mortality and excess morbidity; and (2) it could indicate which investments in non-financial resources and capacity are most likely to lead to future population health benefits.

In this paper we illustrate these issues by building upon economic analyses that were submitted to the WHO HIV Treatment Guideline Review Group on how to monitor the treatment success of people on ART and when to switch to second line regimens. Implicit assumptions made in conventional analyses about the value of non-financial resources are highlighted; as well as the consequences for allocative efficiency if these do not hold. We offer suggestions as to how the real value of non-financial resources can be more accurately reflected in future, when this is likely to be necessary and the implications for decisions to invest in cross-cutting expenditures, such as in health systems strengthening.

### Example: Viral-load informed differentiated care for monitoring people on antiretroviral therapy (ART) in sub-Saharan Africa

Monitoring of people on antiretroviral therapy (ART) is necessary to identify when they are failing treatment and have already developed resistance to drugs, or are at imminent risk of these; and, if HIV is non-suppressed, when they may transmit HIV infection to others. A key role for monitoring is to prompt advice about adherence and to assess the need for a switch to a second line regimen. Monitoring strategies implemented during the roll out of ART in Africa can be broadly clustered into three possible forms of routine patient monitoring: (i) clinically, on the basis of clinical symptoms; (ii) immunologically, using CD4 cell count measurement; and (iii) virologically, using viral load testing. The choice of monitoring strategy also requires decisions about the frequency of monitoring and diagnostic thresholds at which switching ART regimens is recommended.

The cost-effectiveness of alternative approaches to monitoring has been subject to economic analyses, which have evolved as new evidence on effectiveness has become available and prices of testing and of drug regimens change. Viral load monitoring (VLM) is usually clinically preferred and is routinely used in high-income settings, such as Europe and the US, but is expensive and often difficult to implement. For these reasons, it was not recommended by the WHO until it became the preferred monitoring approach in revised 2013 WHO HIV Treatment Guidelines.[7] This was despite an absence of convincing evidence of cost-effectiveness at the time.[4]

In 2015 the HIV Modelling Consortium undertook a comprehensive reassessment of the cost-effectiveness of VLM[5] (box 1). This found that VLM offered some health benefits but these were not large in respect of its costs, such that it was unlikely to be cost-effective if unaccompanied by other changes in clinical care. However, since viral load measurement gives a timely read-out on whether treatment is currently effective (unlike the CD4 count or presence of clinical symptoms) it is in the best position to support ‘differentiated care’–whereby those with elevated VL receive more frequent clinical assessment; but for those with suppressed VL (stable patients) there is simplification of care and a reduction in clinic visit schedules (e.g. to every 6–12 months instead of every 1–2 months). Once the savings from reduced clinic visits for stable patients are incorporated into analyses, these are expected to offset the costs of testing thereby making the scale-up of VLM cost-effective.

**Box 1. The cost-effectiveness of viral-load informed differentiated care of those on ART**

Based upon use of an individual-based stochastic model of heterosexual HIV transmission, natural history, clinical disease and treatment of HIV infection (the ‘HIV Synthesis’ model), calibrated to and simulating the population of Zimbabwe, Phillips et al (2016)[5] evaluated a range of monitoring strategies and predicted outcomes over 20 years to 2035.

In the case of viral-load monitoring (VLM) a strategy was simulated of off-site laboratory monitoring using dried-blood spots (DBS), using the WHO recommended 1,000 RNA copies (cps)/mL threshold, confirmed on a second value after an enhanced adherence intervention, for switching to second line ART. CD4 monitoring strategies were simulated of using either the existing WHO criteria for switching or a new criteria of switching with CD4<200. Clinical monitoring strategies were also included.

In most sub-Saharan African countries, an intervention is only likely to be cost-effective if it delivers health gains (e.g. DALYs-averted) at less than $500 per healthy life year. VLM was estimated to produce some health benefits, but not noticeably more than with CD4 monitoring. If not accompanied by differentiated care, VLM was estimated to deliver health gains at $1,094 per DALY-averted, so was not cost-effective.

However, if measurement of viral load < 1,000 cps ml^-1^ in the last year leads to fewer clinic visits and a corresponding reduction in non-ART programme costs these offset testing costs, leads VLM to deliver health gains at $326 per DALY-averted and to be cost-effective.

This finding relies upon assumptions about how the reduced clinic visits, or the associated cost savings, will be utilized elsewhere and what changes are likely to occur in practice. Given health care workers and the make-up of clinic infrastructures are not fully flexible resources, at least in the short term, the question instead becomes how freed resources will otherwise be employed. If, for example, health care workers simply become under-utilized as a result of differentiated care (e.g. they spend more time on non-health producing activities), the notional, and entirely unrealised, cost-savings from reduced clinic visits are unlikely to be meaningful. If, instead, they shift their attention from routinely seeing individuals with suppressed HIV, for whom the marginal benefits of a clinic visit are low, to giving more attention to patients with more critical need for health care (either within HIV, such as those failing ART or those who wouldn’t otherwise receive access to HIV care; or to those in need of health care with other health conditions) the benefits of freed resources could be very high.

This starkly illustrates that in many cases, whenever resources are not fully flexible to changes in demand for their use, unit costs used in economic evaluations are only a proxy for the value of those resources. Consideration needs to be given to how they would otherwise be used and what the health consequences of this are likely to be. Of course, over extended periods of time (the notional ‘economic long-run’) policy-makers and programme planners have the ability to adjust the stock of non-financial resources (e.g. hire or fire health care workers; restructure clinics) so their availability more closely matches demand and any freed financial resources can be used for other purposes. However, as famously observed by John Maynard Keynes, “in the long run, we are all dead”.[8] Therefore, consideration of the consequences of changing the utilization of fixed resources in the short term is still likely to be required.

It is therefore important to consider the implicit assumptions of economic evaluation studies as conventionally undertaken and when, and how, these assumptions may be relaxed. We extend the work of Phillips et al.[5] to demonstrate these issues.

## Valuing non-financial resources in economic evaluation

### Conventional approach to economic evaluation and their implicit assumptions

Economic evaluation methods aim to support decisions by quantifying the trade-offs between the additional benefits offered by an intervention against any opportunity costs associated with its introduction. Cost-effectiveness analysis (CEA) is the most widely applied approach in health care and usually take as the central objective the generation of measured ‘health’. Health can be captured in units accounting for both mortality and morbidity effects (e.g. QALYs, DALYs-averted), enabling comparisons across diseases. CEA aims to help decision makers answer the question of how available health care resources can best be allocated so as to maximize health across a whole population of concern.

Conceptually, CEA can be viewed as based on a form of constrained optimization. At times, it can be implemented in ways that attempt to reflect directly the sector-wide nature of the resource allocation problem, such as when a mathematical programme is specified to inform which of an array of independent disease programmes and interventions will be funded from within a given set of resources.[9] More typically, a partial approach to CEA is adopted that seeks to identify the cost-effective option from a set of mutually-exclusive alternatives for any given condition (e.g. monitoring options for those on first-line ART) by judging the benefits against the opportunity costs (what will be forgone elsewhere in the health care system). This approach can be viewed as an approximation to the solution of a full mathematical programme making allocation decisions across all health care interventions and should recognize that, if resources are committed to an evaluated intervention, there are interventions for other conditions that also make claims upon limited resources and may be forgone as a consequence.

In principle, the resource constraints included in economic evaluation could be many—including limited health care inputs (health worker time, clinic capacity); and even value judgements, such as equity constraints that all persons with a particular condition are treated equally. It has been recognized for some time that, when specified as a mathematical programme, additional constraints to budget alone (e.g. a maximum number of available bed days) can be included within an analysis,[10] and analyses can indicate where investments in releasing these constraints in the health system would be worthwhile.[11] This approach can also be applied to inform allocation over a whole disease area such as for HIV.[12]

The information requirements for these approaches remain infeasibly large in most cases, however. Typically where economic evaluation to inform actual policy decisions has been applied in practice, more conventional CEA is adopted with the only constraint that is explicitly considered being one of limited financial resources (an available budget). The decision to fund an intervention, j, can then be informed by comparing the health it generates (Δh) and its incremental costs (Δc) to some alternative, and then determining whether the cost per unit of health gained (Δc/Δh) is less than the cost-effectiveness threshold (k), indicating cost-effectiveness:

ΔcΔh<k (1)

The cost-effectiveness threshold reflects the opportunity costs of committing resources to the evaluated intervention in terms of the health those resources could have generated from other priorities forgone as a consequence (i.e. it can be interpreted as the cost per unit of health generated from forgone interventions).

Equivalently, and in some ways more intuitively, the cost-effectiveness of j can be established by directly comparing the health it generates to the health opportunity costs using a net health benefit (NHB) formulation, with positive NHB indicating cost-effectiveness:

NHB = Δh-Δc/k (2)

Based on this method, to inform whether an intervention is likely to increase population health, it is vital that it is appropriately valued in ways that reflect the claims it makes on limited available resources.

The market prices of resources can be used for costing where these are available (e.g. for drugs, technologies). Where they are not, such as for a stay in a public hospital, average costs are typically used that are estimated through combining the change in variable cost (that varies with use of a resource; e.g. food, linen) and an apportioned amount of fixed cost (that does not vary with the output in the short term; e.g. capital costs, wages/salaries of permanent staff). Monetary values are converted to opportunity costs in health terms using the cost-effectiveness threshold. Recent research has estimated cost-effectiveness thresholds by identifying at the margin how health is generated or displaced as health care budgets expand or contract.[13,14]

Although this approach may be adequate in most cases, there is an underlying implicit assumption that may not hold in important ways for some resource allocation decisions. This is that, at the margin, each good or service incurs the same opportunity cost per unit of money (e.g. pound, dollar) spent; or, alternatively, that funds can be released to be spent elsewhere at the same opportunity cost through reduction in the use of resources, as reflected in their unit costs. This assumption is more likely to hold when markets for resource inputs are efficient (i.e. when they are open, competitive and there is full information), leading to what is known as the ‘marginal rate of technical substitution’ (the rate at which one input can be substituted for another, whilst leaving output constant) equalling the ratio of factor prices. For the purposes of economic evaluation, it would mean that it does not matter which type of resource input is expanded or contracted with the delivery of an intervention, because the use value of that resource is entirely reflected in its average unit cost.

It is widely recognized, however, that health care is subject to numerous market failures[15] and, in response, health is primarily a planned sector. For example, wages of skilled health care workers (doctors, nurses) are centrally negotiated and professional bodies restrict the entry of new members. Similarly, decisions as to the make-up of the health system, such as the balance between primary and secondary care, have long-term ramifications well beyond what could have feasibly been initially envisaged.

When opportunity costs are not necessarily equal across resource inputs, this raises the important question of ‘What can be done?’ for the practice of economic evaluation and in the interpretation of results.

### Beyond unit costs: Understanding the value of non-financial resources

CEA conventionally quantifies the value of non-financial resources on the basis of their average unit costs. However, if the use of different types of non-financial resources (e.g. clinic capacity, human resources) has different implications for the production of health, the standard cost-effectiveness framework may need to be adjusted. Specifically, instead of only one cost-effectiveness threshold (k) to reflect opportunity costs of using a limited budget, we may need multiple cost-effectiveness thresholds (ks) depending upon the type of resource is used (or released) in implementing a policy decision.

Assume that the threshold reflecting the use of a flexible health care budget is k_0_ (i.e. at the margin, when financial resources are released, for every k_0_ spent a unit of health is generated). If a particular type of fixed resource (e.g. clinic capacity) is particularly scarce, drawing upon it may result in relatively greater adverse health consequences (higher health opportunity costs) than reallocating the use of the flexible healthcare budget. If this were the case, a lower cost-effectiveness threshold (k_1_) could be applied to the use of that resource that would reflect the higher health opportunity costs (so, k_1_<k_0_). Alternatively, the fixed resource may currently be employed at under-capacity or to relatively unproductive use, in which case the opportunity costs of drawing upon it would be relatively low (so, here, k_1_>k_0_).

Following Van Baal et al.,[16] the standard cost-effectiveness decision rules (1 and 2 above) can be adapted either altering costs of the non-financial resource by the ratio of the cost-effectiveness threshold reflecting its use to that reflecting the use of the flexible health care budget (Eq 3; where $\frac{k0}{k1}$ gives the ratio, or alternatively, the adjustment factor $A\;=\;\frac{k0}{k1}$); or by applying the alternative cost-effectiveness thresholds to the proportion of total costs that correspond to their resource types (Eq 4; where c_0_ represents use of the health budget and c_1_ use a non-financial resource, such as clinic space):

$$\frac{\frac{k0}{k1}\Delta c_{1}+\Delta c_{0}}{\Delta h}<k_{0}\;\text{or}\;\frac{A\cdot \Delta c_{1}+\Delta c_{0}}{\Delta h}<k_{0}$$ (3)

$$\Delta h-(\frac{{\Delta c}_{0}}{k_{0}}+\frac{{\Delta c}_{1}}{k_{1}})$$ (4)

Knowing that different types of resources (flexible financial budgets versus alternative forms of non-financial resources, that are to varying degrees fixed) may be subject to varying opportunity costs is conceptually useful and has real implications for the health generated from alternative policy decisions. However, understanding what those opportunity costs associated with use of fixed non-financial resources are likely to be in reality inevitably requires assessment locally. It is in some ways analogous to marginal costs not necessarily equalling the average costs of resources typically used in costing studies.[17] This causes real challenges for economic evaluations aimed at informing decisions at higher levels (nationally or internationally), since the ramifications of those decisions may vary by locality and may not be very well known anywhere.

An important implication is that recommendations of any cost-effectiveness analyses should be interpreted, on the basis of their many assumptions, as to their appropriateness for a local context. It means there is often likely a trade-off between results that generalize across a country (or even countries) and the extent to which localised circumstances are appropriately reflected.

The stock of non-financial health care inputs can be changed over time (the medium- to long-run) as health care planners expand the capacity of particularly scarce inputs and contract where there is redundant capacity. Any divergence between the cost-effectiveness thresholds may therefore not be expected to last into perpetuity, but could close as decisions are made as to investments in health care inputs—which could even be informed by an understanding that the cost-effectiveness thresholds (opportunity costs of financial and non-financial resources) differ.

### Re-estimating the cost-effectiveness of viral load-informed differentiated care reflecting the non-financial value of resources

Building upon the example outlined in Box 1, we can demonstrate how reflecting the real value of health resources, in terms of how they may be used elsewhere to generate health improvement, can affect the recommendations from economic evaluation studies and has important implications for how such studies are interpreted. For simplification, let us assume there are only three alternatives for how individuals on ART can be monitored: (i) no monitoring/switching; (ii) clinical monitoring; or (iii) viral load monitoring using dried blood spots (VLM-DBS); and each must be applied uniformally across the population.

**Fig 1:**
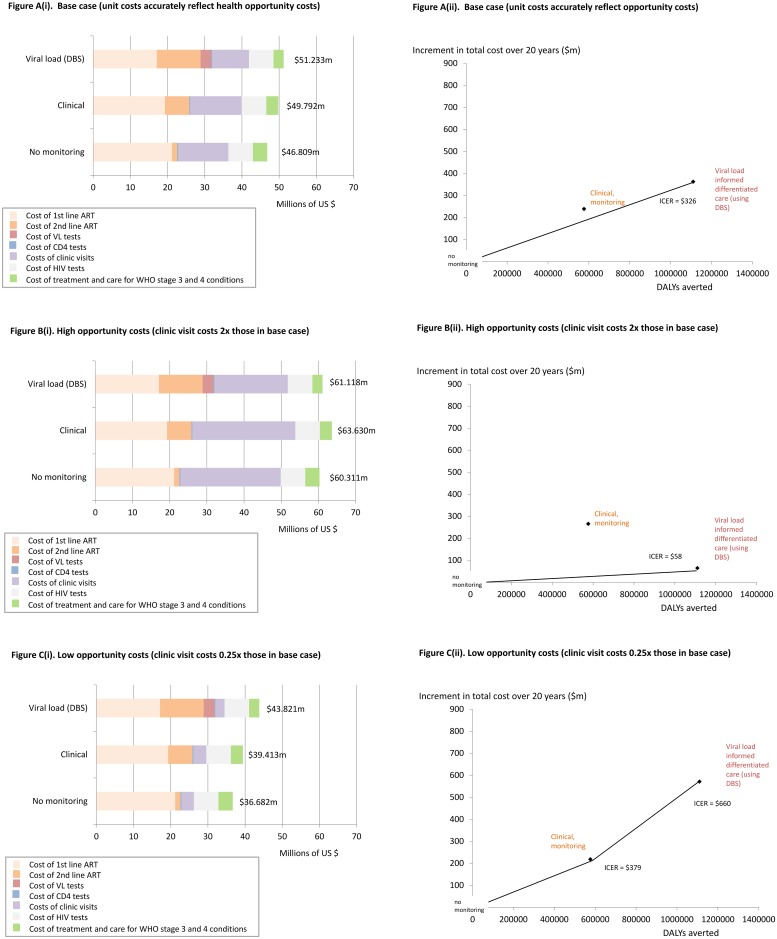
Cost-effectiveness of viral-load informed differentiated care using dried blood spots (VLM-DBS) under alternative assumptions about the opportunity costs of clinic capacity use. Disaggregated costs adjusted for opportunity costs of resources are provided in the left side figures and associated cost-effectiveness planes, showing the incremental cost-per-DALY-averted (ICER) with VLM-DBS, are provided on the right. Under the base case analysis in (A), a cost-effectiveness threshold of $500 is applied to clinic visits and all other costs. The ICER of VLM-DBS is $326. Under the high opportunity scenario (B), a lower cost-effectiveness threshold of $250 is applied to clinic visit costs only and the ICER of VLM-DBS is $58. Under the low opportunity costs scenario (C), a higher cost-effectiveness threshold of $2,000 is applied to clinic visit costs only and the ICER of VLM-DBS is $660.

In the base case analysis (as reported in the published paper), mean costs every 3 months over a 20 year period (2015–34, discounted at 3%) for all components of HIV care (drugs, tests, clinic visits, OI treatment costs) in Zimbabwe would be $46.809m with a strategy of no monitoring/switching to 2^nd^ line ART, $49.792m with clinical monitoring and $51.233m with VLM (DBS) if accompanied by differentiated care. Clinical monitoring is dominated and VLM (DBS) averts a DALY for every $326 spent, which is expected to be cost-effective at a cost-effectiveness threshold of $500 (Fig 1, panel A).

This result relies heavily on reduced clinic visit costs with VLM-DBS off-setting the costs of providing and acting upon VL testing. However, what this means in practice will rely very much on the context in which VLM-DBS is delivered.

Where clinics are at breaking point and face high levels of unmet need, a cost-effectiveness threshold of $500 may not well reflect the competing claims upon their capacity. If the released clinic resources from differentiated care would productively generate health at low cost, a lower cost-effectiveness threshold would be appropriate (e.g. $250, if this was the cost at which clinic freed resources averted a DALY, or an adjustment factor of 2). If, however, clinics were currently under-utilized health opportunity costs associated with use of clinics may be lower and a higher cost-effectiveness threshold (e.g. $2000 per DALY averted, or an adjustment factor of 0.25) may instead be appropriate.

These changes can either be reflected in the traditional cost-per-DALY averted versus cost-effectiveness threshold comparison or in net benefit calculations. Following Eq (3), the traditional cost-per-DALY averted versus a cost-effectiveness threshold approach can be adapted thus:

$$\frac{A\cdot \Delta cvc+\Delta oc}{\Delta DALYs-aveted}<k_{OC}$$

Where cvc are the clinic visit costs, oc are other (non-clinic visit) costs, A is the adjustment factor for clinic to other non-clinic visit costs and *k*_*oc*_ is the threshold applied to other non-clinic visit costs.

In situations in which the health opportunity costs associated with clinic visits are high (e.g. k_cv_ = $250, A = 2), the cost-per-DALY-averted with VLM-DBS would be:

$$\frac{2\Delta cvc+\Delta oc}{\Delta DALYs-averted}$$

$$\frac{64.56m}{1.11m}\;=\;\$\;58\;per\;DALY\;averted,\;versus\;no\;monitoring\;/\;switching$$

Hence VLM-DBS would clearly be cost-effective at a $500 cost-effectiveness threshold (Fig 1, panel B)

In situations in which the health opportunity costs of using clinic capacity are low (e.g. CETcv = $2000, A = 0.25), clinical monitoring is no longer dominated through a range of the cost-effectiveness threshold and the cost-per-DALY-averted with VLM-DBS versus clinical monitoring would be:

$$\frac{0.25\Delta cvc+\Delta oc}{\Delta DALYs-averted}$$

$$\frac{\left(571m-218m\right)}{\left(1.11m-0.57m\right)}\;=\;\$660\;per\;DALY\;averted,\;versus\;clinical\;monitoring$$

Hence, VLM-DBS would no longer be cost-effective at a $500 cost-effectiveness threshold and clinical monitoring would instead be preferred (Fig 1, panel C).

Adapting the net benefit approach to reflect the real value of freed clinic capacity is can similarly be determined by following Eq 4.

We do not propose that cost-effectiveness analyses should routinely apply adjustment factors to account for differing opportunity costs of non-financial resources in base case analyses. This would risk excessive subjectivity, especially because the evidence base on the marginal productivity of such resources is likely to remain limited and depends upon local factors. Instead, the assumption of marginal opportunity costs being equal across use of non-financial resources should be highlighted, so its reasonableness can be assessed in policy deliberations. Where there is sufficient evidence on opportunity costs of non-financial resources the approach can be adopted in scenario (i.e. non-base case) analyses. However, even when there is not, analysts can indicate tipping points of such thresholds that would lead to cost-effectiveness recommendations changing.

## Discussion

In this paper we have highlighted that an implicit assumption in most cost-effectiveness studies—that unit costs of resources suitably reflect the value of those resources in generating health, such that it does not matter which particular resource is utilized or released in the delivery of alternative interventions—may not hold in important ways when those resources are to a degree fixed in the short- to medium- term (i.e. their availability cannot easily be adjusted to changes in demand), and likely to vary by locality. This has potentially important consequences for how economic evaluation is undertaken and the way in which results are used in formulating heath policy and are implemented by programmes in health care delivery.

Although the example was focused on viral load-informed differentiated care for individuals receiving HIV treatment, the implications of unit costs not necessarily well reflecting the health opportunity costs of non-financial resources are potentially wide-ranging, both within HIV and for other forms of health care. Within HIV, the nature of key policy questions has evolved notably in recent times as countries have successfully scaled-up ART to large sections of their populations infected with HIV. Key policy issues have shifted away from primarily *clinical decisions* (e.g. the choice of drug regiment or diagnostic test) and more towards issues of *service delivery*–how to identify those not coming forward for HIV testing, how to ensure long-term retention and viral suppression of those on ART (along the ‘HIV cascade of care’) and how to organize clinics to manage much the high numbers of patients on ART.

The types of interventions typically considered in improving service delivery (e.g. targeted HIV testing, tracing of patients lost to follow-up, task shifting and differentiated care) all have implications for non-financial resources (e.g. staffing, clinic capacities) that cannot easily be scaled up or contracted in response to changes in demand. The availability of these resources is likely to vary by location, even within any one health care system. Careful consideration of the opportunity costs of such non-financial resources is therefore likely to become increasingly important as these issues are tackled. Moreover, HIV services are not delivered in isolation in health care facilities. Programme managers need to content with finding the right mix of heath care inputs to deliver a range of services; so resource considerations will affect how health care across a range of conditions is delivered.

This paper is not intended to advocate a change in the usual approaches to undertaking and using cost-effectiveness analyses in general. Often this will not be necessary; such as when decisions relate primarily to traded commodities (e.g. drugs or devices) or when the use of fixed non-financial resources differs little across alternatives. In other cases, it will simply not be feasible—analysts will continue to have to make the best of limited information in data sparse settings. Instead, our intention is to encourage greater thought in the application and interpretation of what can sometimes be viewed as routine (“off the shelf”) methods; in which there is often greater concern for standardization instead of analyses reflecting the real world contexts in which decisions are made.

It will be intuitive to most people that different types of resources are more or less constrained in different settings (e.g. the relative absence of skilled health care workers in rural areas is widely recognized), but this is sometimes lost in interpreting analyses from models of increasing technical sophistication. For the case of viral-load informed differentiated care, this means the approach is more likely to improve population health when employed in particularly over-stretched clinics, in locations with high other unmet-health care needs. However, it may not be cost-effective in locations in which clinics and staff would be reallocated to relatively less productive activities. The former is probably more likely in most sub-Saharan African settings, hence the analyses for the WHO Guidelines[5] may have underestimated the cost-effectiveness of differentiated care.

Recognizing that the full implications of drawing upon limited non-financial resources are often not well understood and are unlikely to be uniform across settings, even within the same jurisdiction, highlights the value of better evidence on service implementation. The wide collection of disciplines and approaches within the field of implementation science hold particular promise in this regard. Implementation science has gained much traction amongst international HIV service funders in recent years, in particular PEPFAR. However, its links to resource allocation and health policy are often not fully apparent. If it can highlight the value and opportunity costs of drawing upon non-financial resources, this evidence could be used in economic models to better inform policy and programme design. Such studies could include randomized trials of service delivery interventions, in which the use of resource types and interventions forgone as well as delivered are quantified; time-and-motion studies of health care personnel under alternative delivery scenarios; and costing studies of reconfiguring service modalities.

Similarly, economic evaluation models could indicate where greatest uncertainties prevail and additional evidence from implementation science would be of greatest value in reducing the likelihood and consequences of decisions that do not deliver greatest health benefits from within limited resources. Where they show the consequences when opportunity costs differ across types of financial and non-financial resources, such studies can also guide where investments in non-financial resource capacities would be most valuable in generating population health and where disinvestments can be made; in the way it has been proposed mathematical programming could be used.[11] For example, in the case of viral load informed differentiated care, funders investing in viral load testing should probably commit a significant proportion of their funds to ensure the limited non-financial resources involved in delivery (e.g. laboratory staff, transport infrastructures) are also expanded so that other valuable interventions are not foregone, rather than only spend on testing machines and consumables alone.

## Supporting information

S1 File Data and figures Revill et al PLOS ONE submission.xlsx (available at https://doi.org/10.6084/m9.figshare.5632654.v1). Data underlying Fig 1 on the manuscript "Reflecting the real value of health care resources in modelling and cost-effectiveness studies—the example of viral load informed differentiated care", by Paul Revill, Simon Walker, Valentina Cambiano, Andrew Phillips, Mark Sculpher.
(XLSX)
